# Initial management of diabetic ketoacidosis and prognosis according to diabetes type: a French multicentre observational retrospective study

**DOI:** 10.1186/s13613-019-0567-y

**Published:** 2019-08-15

**Authors:** Adrien Balmier, Fadia Dib, Arnaud Serret-Larmande, Etienne De Montmollin, Victorine Pouyet, Benjamin Sztrymf, Bruno Megarbane, Abirami Thiagarajah, Didier Dreyfuss, Jean-Damien Ricard, Damien Roux

**Affiliations:** 1 Intensive Care Unit, Louis Mourier Hospital, AP-HP, 178 rue des Renouillers, 92700 Colombes, France; 2 Department of Anesthesiology and Intensive Care, Bichat-Claude-Bernard Hospital, AP-HP, 75018 Paris, France; 3 INSERM, CIC 1417, F-CRIN, I-REIVAC, Paris, France; 4 AP-HP, Hôpital Cochin, CIC Cochin Pasteur, Paris, France; 5 INSERM, Department of Social Epidemiology, Institut Pierre Louis d’Epidémiologie et de Santé Publique, Sorbonne Université, 75012 Paris, France; 6 Department of Epidemiology, Biostatistics and Clinical Research, Bichat-Claude-Bernard Hospital, Université de Paris, AP-HP, 75018 Paris, France; 7 Intensive Care Unit, Centre Hospitalier de Saint-Denis, Hopital Delafontaine, 93205 Saint Denis, France; 8 INSERM, IAME, UMR 1137, Université de Paris, 75018 Paris, France; 9 Intensive Care Unit, Hôpital René-Dubos, 95300 Pontoise, France; 10 Service de Réanimation polyvalente et surveillance continue, Université Paris Sud, Hôpital Antoine Béclère, AP-HP, 92400 Clamart, France; 11 INSERM U999, 92060 Le Plessis Robinson, France; 12 Department of Medical and Toxicological Critical Care, Lariboisière Hospital, AP-HP, Université de Paris, 75010 Paris, France; 13 INSERM, UMRS-1144, Université de Paris, Paris, France

**Keywords:** Diabetic ketoacidosis, Hypoglycaemia, Hypokalaemia, Insulin, Critical care, Type 1 diabetes, Type 2 diabetes

## Abstract

**Background:**

Guidelines for the management of diabetic ketoacidosis (DKA) do not consider the type of underlying diabetes. We aimed to compare the occurrence of metabolic adverse events and the recovery time for DKA according to diabetes type.

**Methods:**

Multicentre retrospective study conducted at five adult intermediate and intensive care units in Paris and its suburbs, France. All patients admitted for DKA between 2013 and 2014 were included. Patients were grouped and compared according to the underlying type of diabetes into three groups: type 1 diabetes, type 2 or secondary diabetes, and DKA as the first presentation of diabetes. Outcomes of interest were the rate of metabolic complications (hypoglycaemia or hypokalaemia) and the recovery time.

**Results:**

Of 122 patients, 60 (49.2%) had type 1 diabetes, 28 (22.9%) had type 2 or secondary diabetes and 34 (27.9%) presented with DKA as the first presentation of diabetes (newly diagnosed diabetes). Despite having received lower insulin doses, hypoglycaemia was more frequent in patients with type 1 diabetes (76.9%) than in patients with type 2 or secondary diabetes (50.0%) and in patients with newly diagnosed diabetes (54.6%) (*p* = 0.026). In contrast, hypokalaemia was more frequent in the latter group (82.4%) than in patients with type 1 diabetes (57.6%) and type 2 or secondary diabetes (51.9%) (*p* = 0.022). The median recovery times were not significantly different between groups.

**Conclusions:**

Rates of metabolic complications associated with DKA treatment differ significantly according to underlying type of diabetes. Decreasing insulin dose may limit those complications. DKA treatment recommendations should take into account the type of diabetes.

**Electronic supplementary material:**

The online version of this article (10.1186/s13613-019-0567-y) contains supplementary material, which is available to authorized users.

## Background

Diabetic ketoacidosis (DKA) is a severe acute event in diabetes mellitus characterized by dehydration, hyperglycaemia and metabolic acidosis due to ketone hyperproduction. Although historically observed almost exclusively in type 1 diabetes in a context of absolute insulin deficiency, DKA has been increasingly described in type 2 diabetes in the last decades [1–3]. DKA may frequently reveal diabetes mellitus (DKA as first presentation of diabetes).

The pathophysiology of DKA involves severe insulin deficiency and increased levels of counterregulatory hormones (glucagon, catecholamines, cortisol and growth hormone), leading to hyperglycaemia, osmotic diuresis and production of ketone bodies (acetoacetate, 3-beta-hydroxybutyrate and acetone). This results in metabolic acidosis with increased anion gap. Osmotic diuresis explains losses of water, sodium, potassium and phosphate [4, 5].

Diabetic ketoacidosis is, thus, defined by the association of ketonemia ≥ 3 mmol/l or ketonuria ≥ 3+, blood glucose > 11 mmol/l (or known diabetes mellitus), venous plasma bicarbonates (HCO_3_^−^) < 15 mmol/l and/or venous pH < 7.30. The four key points of DKA patient management are (i) admission in a high dependency unit if presence of at least one severity criterium, (ii) rehydration with isotonic saline, (iii) intravenous insulin therapy, and (iv) potassium supplementation [6, 7]. Severity criterium defined by international guidelines include neurological, hemodynamic, pulmonary or biological criterium [6].

Vascular filling followed by insulin therapy is the cornerstone of DKA management. Numerous studies have reported a high incidence of potentially serious adverse events of insulin therapy such as hypoglycaemia and hypokalaemia during DKA management [8–11]. A national survey of DKA management conducted in 2014 in the UK showed rates of 55% of hypokalaemia (at least one episode below 4.0 mmol/l) and 27% of hypoglycaemia (at least one episode) during the 1st day after admission [8].

So far, DKA management in patients with type 2 has been similar to that in patients with type 1 diabetes, although the underlying pathophysiology and clinical profile differ between the two populations, with significant insulin resistance, older age and more prevalent comorbidities in type 2. This undifferentiated approach is not based upon evidence and remains to be demonstrated as appropriate. Since insulin resistance is an important part of type 2 diabetes pathophysiology, we hypothesized that patients with type 2 diabetes are less susceptible to such potentially harmful adverse events. No study has addressed this question. Therefore, in this study, we aimed to compare the metabolic events and the recovery time in adult patients hospitalized for DKA, according to the type of underlying diabetes. We also assessed to what extent the therapeutic management of DKA in the participating centers respected the guidelines [6].

## Methods

### Study design

This is a French multicentre retrospective cohort study of patients admitted for DKA in five adult intensive care units in Paris and its suburb, between January 1, 2013 and December 31, 2014. All participating units were closed units. All medical decisions were, thus, taken by the intensivists in charge. Patients with DKA were identified in the PMSI database (Programme de Médicalisation des Systèmes d’Information) using the ICD 10 codes. All adult patients admitted for DKA as the main diagnoses were included. Patients under 15 years, those with another cause of acidosis and those with hyperglycemic hyperosmolar state were not included. They were, thus, grouped and compared according to the underlying type of diabetes in three groups: patients with known type 1 diabetes (group 1), patients with known type 2 or secondary diabetes (group 2), or patients with DKA as first presentation of diabetes (group 3). Type of diabetes was based on medical files, medical history and treatment regimens. Since it was difficult based on our data to differentiate type 2 diabetes and secondary diabetes, we included these patients in the same group. The study was approved by the Institutional Review Board of Bichat Hospital (approval number: 2018-008).

### Data collection

The following data were recorded from the medical records: demographics and clinical characteristics, the underlying type of diabetes, presence of chronic complications such as microangiopathy (retinopathy, nephropathy or neuropathy), macroangiopathy (cardiovascular disease with coronary arteries, brain or lower limb artery diseases) or chronic renal failure (creatinine clearance below 15 ml/min per 1.73 m^2^), laboratory data (obtained from capillary, blood and urine sampling) and treatments administered within the 48 first hours following hospital admission.

### Outcome variables

Metabolic adverse events during the first 48 h were defined as: hypoglycaemia (at least one episode of blood glucose lower than 4 mmol/l), severe hypoglycaemia (at least one blood glucose lower than 2.9 mmol/l) and hypokalaemia (at least one episode of serum potassium less than 3.5 mmol/l).

Diabetic ketoacidosis resolution time was defined as the interval in hours between the first vascular filling to treat the DKA (performed either in pre-hospital (mobile medical team), in the emergency room or in the ICU) and DKA resolution defined as the occurrence of: the first blood ketone < 0.5 mmol/l, or the first negative ketonuria (0 or 1 cross), or the discharge time from the ICU, which ever came first.

### Biological measurements

The initial blood glucose level was measured either in a venous sample or, if not available, on a capillary glucose test. In the latter case, if the hyperglycaemia measurement threshold was attained, the value of 35 mmol/l was assigned (hyperglycaemia measurement threshold of the capillary glucose meter). Initial ketonemia was the first ketonemia measured either on venous blood in the laboratory or by capillary ketone test. For severity assessment, the anion gap at admission is calculated according to the following formula: (Na^+^ + K^+^) − (Cl^−^ + HCO_3_^−^).

### Statistical analysis

Quantitative variables were presented using median and interquartile ranges, while qualitative variables using numbers and percentages. The three groups were compared on all continuous variables using one-way analysis of variance (ANOVA) tests and Kruskal–Wallis tests as appropriate; they were compared on all qualitative variables using Chi-square and Fisher’s exact tests as appropriate. If significant differences occurred between groups, then paired comparisons were performed using Mann–Whitney *U*-tests and Chi-square tests as appropriate. All *p*-values were 2-sided, and significance was set at *p* < 0.05. Analyses were performed using R version 3.3.0 and SAS (version 9.3, SAS institute Inc., Cary North Carolina).

## Results

### Baseline characteristics

During the study period, 122 patients were admitted to the five participating centers for DKA treatment. Sixty patients (49.2%) had a history of type 1 diabetes, 28 (22.9%) a history of type 2 or secondary diabetes, and 34 (27.9%) had no known history of diabetes. Five pregnant women were included: two patients with a type 1 diabetes and three patients with this episode of DKA revealing a diabetes. Patients were hospitalized in adult intermediate care unit in 61.5% of cases and in adult intensive care unit in 38.5% of cases. Of note, 10 patients between 15 and 18 years old were admitted in our adult units. This is general practice in France where patients older than 15 are considered as adults.

Patient’s characteristics, precipitating causes of DKA and biological results on admission are shown in Table 1. Patients with type 1 diabetes were younger and had a lower weight than other groups (*p* < 0.0001 and *p* < 0.01 as compared to patients with type 2 or secondary diabetes and patients with DKA revealing diabetes, respectively). Patients with type 2 or secondary diabetes had more diabetic chronic complications than other groups (*p* < 0.01 for both). Plasma protein and haemoglobin concentrations (as a marker of dehydration) were higher in patients with newly diagnosed diabetes (*p* < 0.001 and *p* < 0.01 as compared to type 1 diabetes and type 2 or secondary diabetes, respectively). Sex ratio was not different between groups. According to French law, it is not allowed to report patient ethnicity. Hence, this parameter was not recorded.

**Table 1:** Clinical and biological characteristics on admission, according to the type of diabetes

Characteristics at admission	*N*	Total (*n* = 122)	Type 1 diabetes (*n* = 60)	Type 2 or secondary diabetes (*n* = 28)	Newly diagnosed diabetes (*n* = 34)	*p* value
Age (years)	122	43 [24–59]	27 [20–45]	61 [52–70]	42 [27–54]	< *0.001*
Women	122	61 (50.0)	31 (51.7)	13 (46.4)	17 (50.0)	0.901
BMI	82	23.3 [19.5–27.1]	21.3 [19.0–25.5]	23.9 [22.0–30.1]	24.2 [17.5–31.9]	0.136
Weight (kg)	95	65 [55–77]	60 [53–71]	70 [61–80]	69 [55–88]	*0.048*
HbA1C (%)	65	11.1 [8.9–12.8]	10.6 [8.0–12.4]	10.4 [8.9–12.2]	12.7 [11.1–14.0]	*0.019*
Medical background						
Diabetic microangiopathy	114	15 (13.2)	7 (13.2)	8 (28.6)	0 (0)	*0.002*
Diabetic macroangiopathy	114	9 (7.9)	3 (5.6)	6 (22.2)	0 (0)	*0.007*
Chronic renal failure	117	4 (3.4)	0 (0)	4 (14.3)	0 (0)	*0.003*
Cancer	117	7 (6.0)	2 (3.6)	4 (14.3)	1 (3.0)	0.137
Triggering factor						
Poor adherence to treatment	121	49 (40.5)	40 (66.7)	9 (33.3)	0 (0)	< *0.001*
Infection	121	29 (24)	12 (20)	7 (25.9)	10 (24.0)	0.569
Post-surgery	121	1 (0.8)	1 (1.7)	0 (0)	0 (0)	1.000
Other	121	34 (28.1)	20 (33.3)	8 (29.6)	6 (17.6)	0.261
Variables						
HR (/min)	119	110 [95–126]	110 [97–126]	100 [88–120.5]	110 [99–128]	0.141
SBP (mmHg)	119	127 [110–140]	124 [107–135]	133 [110–150]	130 [119–153]	0.065
DBP (mmHg)	119	71 [60–85]	69 [59–81]	74 [57–84]	78 [69–89]	*0.014*
RR (/min)	91	23 [19–29]	24 [20–30]	26 [19–30]	22 [18–25]	0.150
Arterial blood gas						
pH	120	7.16 [7.03–7.24]	7.13 [6.99–7.24]	7.19 [7.11–7.26]	7.13 [7.05–7.24]	0.192
Bicarbonate (mmol/l)	119	6.5 [4.0–10.3]	6.0 [3.1–8.9]	7.8 [4.1–11.7]	6.6 [4.0–10.1]	0.153
PaCO_2_ (mmHg)	117	19 [13–24]	18 [13–22]	20 [15–26]	16 [12–25]	0.092
PaO_2_ (mmHg)	116	120 [98–137]	124 [102–139]	109 [88–126]	122 [104–136]	0.232
Oxygen therapy	117	19 (16.2)	11 (19.0)	10 (40.0)	8 (24.2)	0.126
Intubated patient	115	7 (6.1)	4 (6.9)	2 (8.0)	1 (3.1)	0.775
Blood glucose (mmol/l)	109	32 [24–43.4]	30 [22.3–40]	40.5 [28–50.9]	28.8 [21.6–39.8]	0.261
Ketonuria (cross)	80					0.557
1, *n* (%)		2 (2.5)	0 (0)	1 (5.3)^a^	1 (4.8)^a^	
2, *n* (%)		10 (12.5)	4 (10)	3 (15.8)	3 (14.3)	
3, *n* (%)		19 (23.75)	9 (22.5)	6 (31.6)	4 (19.1)	
4, *n* (%)		49 (61.25)	27 (67.5)	9 (47.4)	13 (61.9)	
Venous blood test						
Na^+^ (mmol/l)	119	133 [128–138]	133 [131–138]	129 [125–137]	134 [128–140]	0.072
K^+^ (mmol/l)	120	4.9 [4.25–5.75]	4.9 [4.3–5.8]	5.1 [4.1–6.1]	4.7 [4.2–5.6]	0.677
Cl^−^ (mmol/l)	117	96 [89–102]	96 [90–103]	91 [83–101]	97 [91–103]	*0.018*
Protein (g/l)	116	79 [68.5–85]	78 [69–85]	68 [62–80]	84 [76–87]	< *0.001*
Urea (mmol/l)	120	8.8 [6.5–17.4]	8.2 [5.7–13.1]	17.3 [9.7–21]	7.4 [5.5–16]	*0.001*
Creatinine (mg/dl)	119	1.44 [0.92–1.99]	1.22 [0.83–1.82]	1.84 [1.36–2.99]	1.22 [0.97–1.98]	*0.012*
Haemoglobin (g/dl)	119	14.7 [12.7–16.0]	14.2 [12.5–16.4]	13.9 [11.4–15.2]	15.6 [14.7–16.5]	*0.005*
Leukocytes (G/l)	98	16.6 [10.1–23.4]	17.2 [10.5–26.0]	16.3 [9.9–23.5]	15.3 [9.2–19.4]	0.169
Lipasemia (UI/l)	59	51 [14–180]	38 [9–123]	34 [12–157]	115 [41–239]	0.856

Data are presented as the median [IQR] or the *n* (%)

BMI: body mass index; HbA1C: glycohemoglobin; HR: heart rate; SBP: systolic blood pressure; DBP: diastolic blood pressure; RR: respiratory rate; PaCO_2_: partial pressure of carbon dioxide in arterial blood; PaO_2_: partial pressure of oxygen in arterial blood

^a^The two patients with only one cross of ketonuria had a ketonemia above 3 mmol/l

### Treatment management

Table 2 presents the characteristics of treatment management (vascular filling and insulin therapy) during the first 48 h. Intravenous insulin therapy was started at the same time as vascular filling in 45.5% of cases. In 47.3% of cases, insulin was started after the beginning of vascular filling. Insulin was administered before the start of filling in 7.3% of cases. Normalized to body weight, patients with type 1 diabetes and patients with newly diagnosed diabetes received a lower insulin dosage (an average of 0.079 U/kg/h and 0.083 U/kg/h, respectively) than patients with type 2 or secondary diabetes (0.104 U/kg/h, *p* < 0.01 and *p* < 0.05, respectively) during the first 6 h of management. In contrast, patients with type 1 diabetes and patients with newly diagnosed diabetes received significantly higher volume of saline during the 1st day as compared to patients with type 2 or secondary diabetes (*p* < 0.05 for both). Although guidelines do not recommend sodium bicarbonate, a few patients received this crystalloid solution (Table 2).

**Table 2:** Treatment according to the type of diabetes

Type of treatment	*N*	Total (*n* = 122)	Type 1 diabetes (*n* = 60)	Type 2 or secondary diabetes (*n* = 28)	Newly diagnosed diabetes (*n* = 34)	*p* value
Vascular filling solution on D1						
Normal saline (0.9% NaCl)	112	112 (100)	54 (100)	25 (100)	33 (100)	
1.4% sodium bicarbonate	108	6 (5.6)	4 (7.8)	1 (4)	1 (3.1)	0.865
4.2% or 8.4% sodium bicarbonate	108	7 (6.5)	3 (5.9)	3 (12)	1 (3.1)	0.464
Colloid	108	1 (0.9)	1 (2.0)	0 (0)	0 (0)	1.000
Volume of normal saline infused on D1 (ml)	112	3500 [1250–5000]	3750 [2000–6000]	2000 [1000–3500]	4000 [1500–5000]	*0.013*
IV insulin treatment						
Bolus	111	30 (27.0)	13 (24.5)	9 (36)	8 (24.2)	0.517
Initial insulin infusion rate (IU/h)	112	7 [5–10]	6 [5–10]	9 [5–10]	7 [5–10]	0.356
Cumulative insulin dose (IU), per period						
Cumulative amount on D1#1	107	30 [20–50]	25 [16–42]	40 [28–60]	35 [21–50]	*0.006*
Cumulative amount on D1#2	107	42 [30–60]	42 [30–60]	60 [25–65]	40 [34–60]	0.517
Cumulative amount on D1#3	107	36 [26–60]	36 [23–50]	55 [27–73]	36 [30–60]	0.254
Cumulative amount on D1#4	104	30 [18–51]	25 [18–40]	28 [16–75.5]	36 [18–49]	0.389
Cumulative amount on D2#1	96	44 [28–68]	36 [25–60]	46 [27–108]	55 [36–70]	0.211
Cumulative amount on D2#2	89	40 [30–60]	37 [24–52]	44 [33–106]	48 [32–63]	0.276

Data are presented as the median [IQR] or the *n* (%)

D1: first 24 h after filling start; U: insulin unity; D1#1: from H0 to H6; D1#2: from H6 to H12; DI#3: from H12 to H18; DI#4: from H18 to H24; D2#1: from H24 to H36; D2#2: from H36 to H48

### Metabolic events and mortality

The rate of hypoglycaemia and profound hypoglycaemia during the first 48 h was significantly higher among patients with type 1 diabetes as compared to patients with type 2 or secondary diabetes (76.9% vs. 50.0%, *p* = 0.016 and 40.4% vs. 18.5%; *p* = 0.032, respectively) and to patients with newly diagnosed diabetes (76.9% vs. 54.5%; *p* = 0.032 and 40.4% vs. 18.2%; *p* = 0.049, respectively). Twenty-eight (82.4%) patients with DKA as revealing presentation of diabetes had at least one episode of hypokalaemia during the first 48 h of management, an incidence significantly higher than the one observed in patients with type 1 diabetes (*n* = 34, 57.6%, *p* = 0.015) and type 2 or secondary diabetes (*n* = 14, 51.9%, *p* = 0.011). Hypophosphatemia below 0.3 mmol/l was observed in 24 patients (21.4%) without statistical differences between groups. Exhaustive report of metabolic events is depicted in Additional file 1: Table S1. Overall, these complications mandated intravenous or oral supplementation of glucose, potassium or phosphate. Insulin infusion has to be temporarily stopped in 23.4% of the patients. However, no neurological or cardiovascular effects of these metabolic complications were reported in medical files.

One patient with type 2 diabetes died; he was a 74-year-old man with a history of chronic calcific pancreatitis and pleural mesothelioma.

### Resolution time

The resolution times were not statistically different between groups (type 1 diabetes: 16 h [IQR = 11–26]; type 2 or secondary diabetes: 14 h [IQR = 8–23] and patients with newly diagnosed diabetes: 16 h [IQR = 12–28]; *N* = 91).

### Adequacy to UK recommendations regarding admission

Since no French recommendations regarding DKA management were published, we used the UK guidelines to evaluate the adequacy of admission in intermediate or intensive care unit using severity criteria [6]. One-hundred and twenty-one patients (99.2%) had at least one intermediate and intensive care unit admission criteria according to the UK guidelines. The details of these criteria are presented for the entire population in Table 3.

**Table 3:** Criteria for admission to the intensive care unit according to the 2011 British guidelines, by type of diabetes

Initial characteristics	N	Total (*n* = 122)	Type 1 diabetes (*n* = 60)	Type 2 or secondary diabetes (*n* = 28)	Newly diagnosed diabetes (*n* = 34)
Glasgow Coma Scale < 12	120	13 (10.8)	8 (13.3)	2 (7.4)	3 (9.1)
SBP < 90 mmHg	119	14 (11.8)	9 (15.8)	4 (14.3)	1 (2.9)
HR < 60 or > 100 bpm	119	81 (68.1)	41 (71.9)	16 (57.1)	24 (70.6)
SpO_2_ < 92%	120	9 (7.5)	3 (5.2)	1 (3.6)	5 (14.7)
pH < 7.10	120	43 (35.8)	26 (43.3)	6 (21.4)	11 (32.4)
Bicarbonate < 5 mmol/l	119	40 (33.6)	23 (38.3)	7 (25.9)	10 (31.2)
Serum potassium < 3.5 mmol/l	120	9 (7.5)	4 (6.8)	3 (11.1)	2 (5.8)
Anion gap > 16 mmol/l	114	112 (98.2)	56 (100)	26 (96.3)	30 (96.8)
Blood ketone concentration > 6 mmol/l	40	13 (32.5)	6 (28.6)	2 (33.3)	5 (38.5)
At least one criterium present	122	121 (99.2)	60 (100)	27 (96.4)	34 (100)

Data are presented as the *n* (%)

SBP: systolic blood pressure; HR: heart rate; bpm: beat per minute; SpO_2_: blood oxygen saturation

## Discussion

This study reports management and complications of DKA depending of underlying type of diabetes, including newly diagnosed diabetes. Its main findings can be summarized in three points. First, we confirm that there is a significant prevalence of patients with type 2 or secondary diabetes with DKA hospitalized in intermediate and intensive care units; patients with type 2 or secondary diabetes being older with higher body weight and more chronic complications of diabetes. Second, we notice a higher incidence of hypoglycaemic events during DKA treatment in patients with type 1 diabetes despite being treated with lower insulin doses; observation compatible with the hypothesis of a lower level of insulin resistance in patients with type 1 diabetes as compared to patients with type 2 diabetes. And third, we observe a higher incidence of hypokalaemia in patients with newly diagnosed diabetes, in a context of more severe dehydration on admission (higher haemoglobin and plasma protein concentrations).

Despite the increasing occurrence in patients with type 2 or secondary diabetes and differences in pathophysiology between type 1 diabetes and type 2 or secondary diabetes, guidelines for management of DKA do not distinguish these patients [6]. In view of the differences observed in our study, the appropriateness of currently recommended management of DKA, not considering the type of underlying diabetes, may be questioned.

Many previous studies have shown that DKA occurs increasingly in patients with type 2 diabetes [1, 12, 13]. In our cohort, the proportion of type 2 or secondary diabetes among all DKA patients (23%) was similar to other recent publications, confirming in France what was previously observed in the US and the UK. Accordingly, Newton et al. [1] found 21.7% of type 2 diabetes in a cohort of 138 patients admitted for moderate to severe DKA. Barski et al. [12] observed 17.4% of patients with type 2 diabetes in their cohort of 201 patients. As observed in other studies, the characteristics of these patients differed significantly from those with type 1 diabetes: patients with type 2 or secondary diabetes were older, had more chronic complications of diabetes (microangiopathy or macroangiopathy, chronic renal failure), had higher incidence of cancers and higher body mass index.

Precipitating causes of DKA confirmed what was already observed worldwide with a predominance of poor adherence to treatment in patients with type 1 diabetes and a significant role of infection which concerned about 25% of the patients with DKA [14].

Patients in all three groups had comparable blood glucose on admission. Although patients with type 1 diabetes received lower insulin doses than patients with type 2 or secondary diabetes and lower than those recommended in the UK guidelines [6], they had more episodes of hypoglycaemia (including profound hypoglycaemia). In contrast, patients with type 2 or secondary diabetes received an initial dose of insulin in accordance with the UK recommendations. This result suggests that, in our units, clinicians already considered the type of underlying diabetes in the management of DKA. In addition, it suggests that a decreased dose of insulin, either at its initiation or when glucose level decreased below a determined threshold as proposed by other authors [14], could help in preventing this frequent complication.

Interestingly, a non-inferiority randomized controlled trial (RCT) comparing reduced dose of insulin (0.05 UI/kg/h) with standard recommended dose (0.10 UI/kg/h) in children with DKA including almost exclusively patients with type 1 diabetes reported significantly less metabolic events in the group receiving the reduced dose of insulin, without altering the recovery time [15]. It is reasonable to think that a similar effect would be observed in a DKA episode among adult patients with type 1 diabetes.

The nationwide survey on the management of DKA in the UK by Dhatariya et al. [8] did not differentiate patients by type of diabetes. Comparing our study populations at admission, it reveals that our patients were slightly more severe with higher blood glucose level (32 vs. 28.7 mmol/l) and lower bicarbonate concentration (6.5 vs. 11.3 mmol/l). In the study by Dhatariya et al., 27.6% of patients had at least one episode of hypoglycaemia during the first 24 h. The threshold for diagnosing hypoglycaemia was not clearly indicated. In our study, 51.4 and 20.5% of patients had at least one episode of blood glucose lower than 4 and 2.9 mmol/l, respectively. Similarly, while Dhatariya et al. reported that 55% of patients developed an hypokalaemia during the first 24 h, we observed that 63.3% of our patients presented at least one episode of serum potassium less than 3.5 mmol/l during the first 48 h of management [8]. Overall, it appears that the rate of metabolic events during DKA care remains high.

The recovery time in our study was quite similar to the one reported by Dhatariya et al. [8] (16.0 vs. 18.7 h). In our study, the recovery time was not different between the three groups. This could be explained either by the fact that patients with type 2 or secondary diabetes received more insulin, compensating for the increased insulin resistance of this population, or because a low dose of insulin is sufficient to block lipolysis, release of free fatty acids and production of ketone bodies. This is concordant with the absence of difference in recovery time observed in type 1 diabetes paediatric patients in the RCT of Nallasamy et al. [15] despite halved dose in the experimental group.

The results reported in the group of patients developing a DKA as first presentation of diabetes also bring new insights. This group of newly diagnosed diabetes is heterogeneous, including both young and older patients. Since no prior history of diabetes was known in these patients, DKA evolved probably for a longer time before diagnosis. This is suggested by the higher severity of extracellular dehydration than in the other two groups (significantly higher plasma protein and haemoglobin concentrations). It can be argued that water and sodium loss correlated with potassium losses, explaining the higher incidence of hypokalaemia among patients of this group. In a paediatric study by Lopes et al. [16], there were also a greater number of hypokalaemia episodes in the group of patients with newly diagnosed diabetes.

All but one patients of the present study were correctly referred to an ICU in regard to UK recommendations (at least one criterium of severity) [6]. The elevation of the anion gap beyond 16 mmol/l was the most frequent factor leading to ICU hospitalization (98.2% of patients). However, since elevated anion gap above 16 mmol/l mainly confirms the presence of ketone bodies and does not systematically reflect the severity of DKA, we believe this criterion is not useful to identify the most severe patients; it is rather a diagnostic criterion of the DKA. A threshold greater than 16 would probably be more discriminating. Unfortunately, since a large proportion of patients admitted in our ICUs were transferred from emergency department of other hospital or directly from home (using French mobile emergency medical service), it is not possible to evaluate the total number of patients hospitalized for a DKA, and thus the proportion of patients referred to our units.

In our study, a few patients received intravenous hyperosmolar sodium bicarbonate. This treatment is not recommended in international guidelines and should be avoided since it may be associated with an increased risk of cerebral oedema [17, 18]. It underlines the need for French recommendations on DKA management that have not been produced until now.

This study has limitations. First and foremost, it is retrospective. If type 1 diabetes was well documented in patients’ files, it was difficult to differentiate accurately type 2 diabetes from secondary diabetes. In this context we decided to include these patients in a single group (type 2 and secondary diabetes). Second, practices and monitoring were not strictly similar among study centers, and patients’ files were either computerized or paper based, thus conferring heterogeneity of data availability. Third, due to sample size limitations, we were unable to perform multivariate analysis to fully investigate the role of the type of diabetes (with adjustment for confounding variables) in the occurrence of hypoglycaemia following management of DKA.

## Conclusion

We found a greater incidence of hypoglycaemia in patients with type 1 diabetes during DKA management, as compared to patients with type 2 or secondary diabetes, despite lower insulin doses. Patients with unknown diabetes (DKA as first presentation of diabetes) appeared to have more severe extracellular dehydration and developed more hypokalaemia episodes than DKA patients with previously diagnosed diabetes. Since no difference was observed for the time to achieve resolution of DKA, reducing insulin doses in patients with type 1 diabetes as well as in patients with DKA as the first presentation of diabetes would likely help prevent metabolic complications. Randomized controlled studies comparing reduced insulin doses to standard care in these patients are required to improve DKA care.

## Additional file

**Additional file 1: Table S1.** Metabolic complications (hypoglycaemia, hypokalaemia, hypophosphatemia) according to the type of diabetes.

## Data Availability

All data are conserved at the “Unite de Recherche Clinique” at Bichat hospital, Paris, under the supervision of Dr Fadia Dib.
