# Supplementary material for: Initial management of diabetic ketoacidosis and prognosis according to diabetes type: a French multicentre observational retrospective study
Source: Ann Intensive Care. 2019 Aug 15;9:91. doi: 10.1186/s13613-019-0567-y (PMC6695456; doi:10.1186/s13613-019-0567-y)
Supplement: Supplementary file 1 — Additional file 1: Table S1. Metabolic complications (hypoglycaemia, hypokalaemia, hypophosphatemia) according to the type of diabetes. [file 13613_2019_567_MOESM1_ESM.docx]

Additional file

Initial management of diabetic ketoacidosis and prognosis according to diabetes type: a French multicentre observational retrospective study.

Adrien Balmier, MD^1,2^, Fadia Dib, MD^3,4,5^, Arnaud Serret-Larmande^3^, Etienne De Montmollin, MD^6,7^, Victorine Pouyet, MD^8^, Benjamin Sztrymf, MD, PhD^9,10^, Bruno Megarbane, MD, PhD^11,12^, Abirami Thiagarajah, MD^1,8^, Didier Dreyfuss, MD^1,7^, Jean-Damien Ricard, MD, PhD^1,7^, Damien Roux, MD, PhD^1,7^

**Table S1: Metabolic complications (hypoglycaemia, hypokalaemia, hypophosphatemia) according to the type of diabetes.**

| **Complications** | **N** | **Total**  **(n = 122)** | **Type 1 diabetes (n = 60)** | **Type 2 or secondary diabetes (n = 28)** | **Newly-diagnosed diabetes (n = 34)** | **P value** |
| --- | --- | --- | --- | --- | --- | --- |
| **Death, n (%)** | 121 | 1 (0.8) | 0 (0) | 1 (3.7) | 0 (0) | 0.220 |
| **Hypoglycaemia** |  |  |  |  |  |  |
| Lowest glucose level on D1 (in mmol/l) | 114 | 3.7 [2.9-6.3] | 3.6 [2.8-4.9] | 5.2 [3.4-6.3] | 4.7 [3.2-6.9] | 0.777 |
| Lowest glucose level on D2 (in mmol/l) | 104 | 4.3 [3.3-6.3] | 3.8 [3.1-5.7] | 4.5 [3.6-5.9] | 4.8 [3.3-6.8] | 0.277 |
| Lowest glucose level on D1 and D2 (in mmol/l) | 114 | 3.5 [2.8-4.8] | 3.1 [2.5-3.9] | 3.6 [3.1-5.2] | 3.5 [2.9-6.2] | **0.028** |
| Nb of episodes with glucose concentration < 4 mmol/l on D1, mean (SD) | 111 | 1.0 (1.3) | 1.3 (1.5) | 0.7 (1.1) | 0.6 (0.8) | **0.035** |
| Nb of episodes with glucose concentration < 2.9 mmol/l on D1, mean (SD) | 112 | 0.3 (0.7) | 0.4 (0.8) | 0.2 (0.6) | 0.2 (0.6) | 0.416 |
| Nb of episodes with glucose concentration < 4 mmol/l, mean (SD) | 100 | 0.8 (1.2) | 1.0 (1.2) | 0.5 (0.7) | 0.8 (1.4) | 0.240 |
| Nb of episodes with glucose concentration < 2.9 mmol/l on D2, mean (SD) | 100 | 0.15 (0.5) | 0.3 (0.6) | 0.04 (0.2) | 0.03 (0.18) | **0.026** |
| Nb of episodes with glucose concentration < 4 mmol/l on D1 and D2, mean (SD) | 111 | 1.7 (1.8) | 2.1 (1.9) | 1.2 (1.5) | 1.3 (1.6) | **0.026** |
| Nb of episodes with glucose concentration < 2.9 mmol/l on D1 and D2, mean (SD) | 112 | 0.4 (0.8) | 0.6 (1.0) | 0.3 (0.7) | 0.2 (0.8) | **0.039** |
| At least one episode < 4 mmol/l on D1, n (%) | 111 | 57 (51.4) | 33 (63.5) | 10 (38.5) | 14 (42.4) | 0.054 |
| At least one episode < 2.9 mmol/l on D1, n (%) | 112 | 23 (20.5) | 14 (26.9) | 4 (14.8) | 5 (15.1) | 0.297 |
| At least one episode < 4 mmol/l on D2, n (%) | 100 | 44 (44.0) | 25 (54.4) | 8 (33.3) | 11 (36.7) | 0.152 |
| At least one episode < 2.9 mmol/l on D2, n (%) | 100 | 12 (12.0) | 10 (21.7) | 1 (4.2) | 1 (3.3) | **0.033** |
| At least one episode < 4 mmol/l on D1 and D2, n (%) | 111 | 71 (64.0) | 40 (76.9) | 13 (50.0) | 18 (54.6) | **0.026** |
| At least one episode < 2.9 mmol/l on D1 and D2, n (%) | 111 | 32 (28.6) | 21 (40.4) | 5 (18.5) | 6 (18.2) | **0.036** |
| **Hypokalaemia** | | | | | | |
| Lowest potassium level on D1 (in mmol/l) | 119 | 3.5 [3.2-3.8] | 3.6 [3.3-3.9] | 3.7 [3.3-4.0] | 3.3 [2.9-3.5] | **<0.001** |
| Lowest potassium level on D2 (in mmol/l) | 111 | 3.4 [3.1-3.9] | 3.5 [3.1-3.9] | 3.8 [3.2-4.0] | 3.1 [3.0-3.6] | **0.012** |
| Lowest potassium level on D1 and D2 (in mmol/l) | 120 | 3.3 [3.0-3.7] | 3.4 [3.1-3.7] | 3.4 [3.1-3.9] | 3.1 [2.8-3.3] | **<0.001** |
| Nb of episodes with serum potassium < 3.5 mmol/l on D1, mean (SD) | 119 | 0.47 (0.50) | 0.38 (0.49) | 0.37 (0.49) | 0.71 (0.46) | **0.005** |
| Nb of episodes with serum potassium < 3.5 mmol/l on D2, mean (SD) | 111 | 0.52 (0.50) | 0.49 (0.50) | 0.4 (0.50) | 0.67 (0.48) | 0.109 |
| Nb of episodes with serum potassium < 3.5 mmol/l on D1 and D2, mean (SD) | 120 | 0.63 (0.48) | 0.58 (0.50) | 0.52 (0.51) | 0.82 (0.39) | **0.023** |
| At least one episode < 3.5 mmol/l on D1 and D2, n (%) | 120 | 76 (63.3) | 34 (57.6) | 14 (51.9) | 28 (82.4) | **0.022** |
| **Hypophosphatemia** |  |  |  |  |  |  |
| Lowest phosphate level on D1 (in mmol/l) | 107 | 0.56 [0.33-0.93] | 0.61 [0.4-0.89] | 0.65 [0.34-1.09] | 0.46 [0.3-0.81] | 0.267 |
| Lowest phosphate level on D2 (in mmol/l) | 85 | 0.60 [0.41-0.84] | 0.59 [0.41-0.86] | 0.65 [0.48-0.79] | 0.56 [0.41-0.86] | 0.960 |
| Lowest phosphate level on D1 and D2 (in mmol/l) | 104 | 0.49 [0.31-0.8] | 0.56 [0.33-0.81] | 0.49 [0.32-0.79] | 0.44 [0.29-0.76] | 0.588 |
| At least one episode < 0.3 mmol/l on D1, n (%) | 107 | 21 (19.6) | 9 (18.0) | 4 (16.7) | 8 (24.2) | 0.738 |
| At least one episode < 0.3 mmol/l on D2, n (%) | 85 | 9 (10.6) | 5 (12.5) | 1 (5.3) | 3 (11.6) | 0.813 |
| At least one episode < 0.3 mmol/l on D1 and D2, n (%) | 112 | 24 (21.4) | 12 (21.8) | 4 (16.7) | 8 (24.2) | 0.785 |

Data are presented as the median [IQR] or the n (%) or mean (SD). Nb = number; D1 = first 24 hours after vascular filling start; D2 = next 24 hours after D1.
